# PIMMS-Dash: Accessible analysis, interrogation, and visualisation of high-throughput transposon insertion sequencing (TIS) data

**DOI:** 10.1016/j.csbj.2024.10.025

**Published:** 2024-10-18

**Authors:** Adam M. Blanchard, Adam Taylor, Andrew Warry, Freya Shephard, Alice Curwen, James A. Leigh, Richard D. Emes, Sharon A. Egan

**Affiliations:** a University of Nottingham, School of Veterinary Medicine and Science, Leicestershire LE12 5RD, United Kingdom; b University of Nottingham, Digital Research Services, Nottingham NG7 2RD, United Kingdom; c Nottingham Trent University, Nottingham NG1 4FQ, United Kingdom

**Keywords:** Transposon Mutagenesis, TIS

## Abstract

**Motivation:**

Current methods for visualising and interrogating high-throughput transposon insertion mutagenesis sequencing (TIS) data requires a significant time investment in learning bioinformatics, often producing static figures that do not facilitate real time analysis.

**Summary:**

We have created an accessible web-based browser tool for visualisation and downstream analysis of high-throughput TIS data results generated by the PIMMS analysis pipeline. This includes multiple interactive and sortable tables to aid the user to identify genes of interest, enabling the user to gain a greater understanding of the genes contributing to fitness in their experimental work. PIMMS-Dash permits researchers, with any level of bioinformatics knowledge, to interrogate data sets and generate publication quality figures.

**Availability:**

PIMMS-Dash is freely available and is accessible online at https://pimms-dashboard-uon.azurewebsites.net and a Docker containerised version is available at https://github.com/Streptococcal-Research-Group/PIMMS-Dash to run locally.

## 1 Introduction

Transposon/insertional mutagenesis is a powerful tool frequently used in microbiology to elucidate genes that contribute to fitness (growth/survival). Disrupting genes by random insertional mutagenesis, provides a resource to achieve a greater understanding of how bacterial genotypes contribute to their observed phenotypes. Approaches involve the construction of mutant libraries containing a high-density of insertions before the library is exposed to the selective condition. Frequency of mutations can then be compared before and after exposure to the selective environment or between selective and non-selective environments. This is achieved by high throughput sequencing, focussed on the genome/insertion junctions, allowing for the quantification of conditionally essential sequences within the selective condition.

The main laboratory methods for high-throughput mutagenesis mapping are Insertion Sequencing (INSeq) [1], Transposon Insertion Sequencing (TN-Seq) [2], High-throughput Insertion Tracking by deep Sequencing (HITS) [3], Transposon-Directed Insertion site Sequencing (TraDIS) [4] and Pragmatic Insertion Mutant Mapping System (PIMMS) [5], [6]. Each approach is similar, however there are subtle differences in applicable transposons or insertional elements, mutant library preparation, sequencing approaches and software tools used for bioinformatics analysis. There are numerous bioinformatic tools for analysing the raw sequence data for the multiple different experimental approaches which are summarised elsewhere (Cain et al., 2020) but currently there are no tools which offer accessible statistical analysis and visualisation that can be finely tuned for each dataset.

To complement the currently available bioinformatics analysis tools we have developed PIMMS-Dash, a complementary collection of tools working from a simple browser based interface allowing for quick and easy downstream analysis, statistical evaluation and visualisation of insertional mutation mapping sequence data.

## 2 Implementation

The PIMMS-Dashboard was developed in Python version 3.9 using the IDE Pycharm version 2020.2. The app makes use of following external python packages; numpy (v1.20.1) [7], pandas (v1.2.3) [8], plotly (v4.14.3) [9], dash (v1.19.0) (https://dash.plotly.com). Additionally, DESeq processing is done using R version 4.0.4 and the package DESeq2 (v3.14) [10]. Interaction between python and R scripts is handled with the package Rpy2 (v3.4.4) (https://github.com/rpy2/rpy2/). The web app is hosted on the Azure App Service Plan and can be accessed here [https://pimms-dashboard-uon.azurewebsites.net/]. Docker (v20.10.7) was used to containerise the application and push the container image to the Azure container registry.

## 3 Usage

**Fig. 1:**
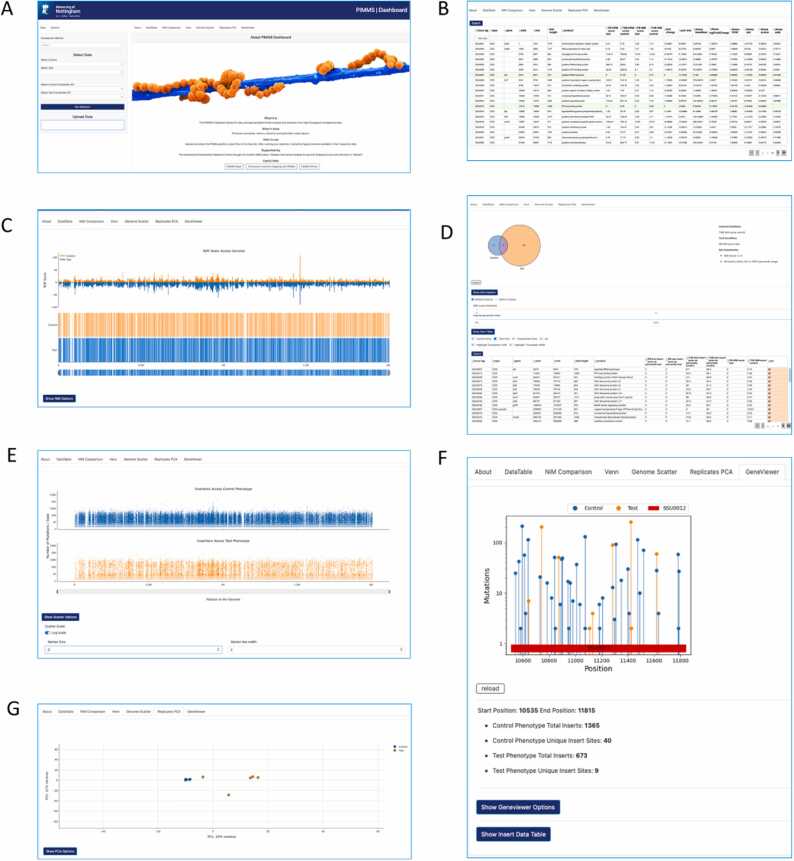
**Figure One Demonstration of the available tabs in PIMMS-Dash**: A) Landing page for uploading data and applying settings B) Normalised data to directly compare between test and control mutant libraries and insertional positions. C) Data table with statistical analysis. D) Venn page to identify and subset essential genes. E) Genome Scatter page to visualise regions of genome which lacks any mutations or identify areas of high saturation. F) Gene Viewer page to interrogate the localisation of the insertions at a CDS level. G) Scatter plot to visualise the (dis)similarity of the pools.

There are two sets of example data provided on the web app. The respective “control” and “test” files can be selected in the left control panel alongside their “coordinate-gff files” and run through the dashboard. The PIMMS-Dashboard pre-loaded datasets are from a high-throughput insertional mutagenesis sequencing experiment comparing Streptococcus suis (P1/7) following growth in laboratory medium (Todd Hewitt broth) and pig serum (Accession number PRJNA1169786). Users can also upload their own csv and gff files which can be generated using PIMMS2 (https://github.com/Streptococcal-Research-Group/PIMMS2). The new data can be easily uploaded using the drag and drop option on the home screen (Fig. 1A). This will accept files from the PIMMS data analysis pipeline [5] where a directory is created containing the files which are needed for the dashboard. The data can be generated from any high-throughput mutagenesis experiment including, but not limited to, TraDIS [4], Tn-Seq [2] and HITS [1]. Any uploaded data is only available to the current browser session and does not become publicly available. The general dashboard options can be found in the options tab on the left control panel. These include plot configurations for the visualisation tabs and the ability to toggle DESeq2 processing on or off. We recommend that the default outlier removal in DESeq2 is left enabled.

After loading data and selecting the analysis options, the user can work through the tabs to see different results. The six available tabs which allow for the user to visualise the uploaded data table to check its integrity. The data table tab is a replication of the uploaded csv file, showing the information of the annotated genome, including the NRMs for each sample. There are also additional columns produced from the DESeq2 [10] module should it have been activated in the options. The output of these data provides an indication of the log2 fold change of the number of insertions for each coding sequence between the two conditions, determining the relative fitness (Fig. 1B). This is important as some mutations could be lost but not become essential due to compensation within a metabolic pathway. A base mean is also produced along with a raw p-value and multiple comparison corrected p-value using Benjamin-Hochberg correction. Each column can be sorted and searched using the column header and a radio button is next to each column to select a gene of interest. This will activate the Gene Viewer tab to visualise insertion positions and compare phenotypic changes.

The NIM Comparison tab allows users to visualise the saturation of insertions across the genomes, between each condition (Fig. 1C). NIM is Normalized Insertions Mapped (total unique insertions mapped/Length of gene in Kb)/(total insertions mapped/106) or the additional NRM option which are Normalized Reads Mapped (total number of reads/length of gene in Kb)/(total mapped read count/106). These provide an indication of the disruption of a given gene in comparison to others within the population and also takes into account the variability of the number of mapped sequence reads for each experiment. The function of this tab is to allow the user to quickly assess if there are any regions of the genome which have acquired a disproportionate number of mutations, or “hot spots”. This is a useful quality control step to ensure the mutations are random and has no negative impact on the analysis from poor mutation saturation, PCR or sequence library bias.

The Venn tab enables users to identify essential of fitness associated genes which are shared or unique to the conditions tested and export this subset list of genes from a chosen intersect (Fig. 1D). There are additional options to allow further filtering of the results. The sliders can be moved to increase the NIM score to include rare insertional events, or the percentile slider can ignore insertions which appear in the first or final percent of a gene. This can be important to remove insertions which would not disrupt a N or C terminus amino acid and change the function of the gene.

The Genome Scatter tab produces an interactive figure where the user can zoom in on regions of the genome to investigate larger areas of essential genes and see if they are represented in both conditions (Fig. 1E). The Replicates tab produces a PCA which offers the user a method of quality control to see if the replicates cluster as would be expected for the two conditions (Fig. 1F). Finally, the GeneViewer tab enables finer scale assessment of the insertions detected in a specific gene (Fig. 1G). A specific gene of interest can be selected in the data table tab which will show each unique insertion point and number of insertions in the GeneViewer tab. This is helpful if used in conjunction with the Venn percentile sliders.

## 4 Conclusion

Exploration and analysis of high throughput still remains a daunting task for a bioinformatics novice. PIMMS2 and PIMMS-Dash offers an accessible approach to insertion mapping data analysis with the emphasis on quick and easy statistical analysis and visualisation of results to a publication ready standard.

## CRediT authorship contribution statement

**Adam Blanchard:** Writing – review & editing, Writing – original draft, Validation, Supervision, Project administration, Methodology, Conceptualization. **Sharon A. Egan:** Writing – review & editing, Writing – original draft, Supervision, Funding acquisition, Conceptualization. **James A. Leigh:** Writing – review & editing, Writing – original draft, Supervision, Project administration, Investigation, Funding acquisition, Conceptualization. **Richard D. Emes:** Writing – review & editing, Supervision, Conceptualization. **Freya Shephard:** Writing – review & editing, Supervision, Formal analysis, Data curation. **Alice Curwen:** Writing – review & editing, Investigation, Formal analysis, Data curation. **Adam Taylor:** Writing – original draft, Validation, Software, Methodology. **Andrew Warry:** Software, Project administration, Conceptualization.

## Funding Information

This work was funded in part by the International Development Research Centre (IDRC) [Ref: 109056] and the Biotechnology and Biological Sciences Research Council (BBSRC) [Ref: BB/T001933/1]. Alice Curwen was supported by a BBSRC Doctoral Training Programme (DTP) PhD studentship.

## Author Statement

Each named author has substantially contributed to conducting the underlying research and drafting this manuscript. Additionally, the named authors have declared no conflict of interest, financial or otherwise. All authors approved the submission to CSBJ. The manuscript has not been submitted to another journal prior to this submission.

## Declaration of Competing Interest

None.
